# Supplementary material for: Patterns of Intron Gain and Loss in Fungi
Source: PLoS Biol. 2004 Nov 30;2(12):e422. doi: 10.1371/journal.pbio.0020422 (PMC532390; doi:10.1371/journal.pbio.0020422)
Supplement: Table S1 — Also available at http://genes.mit.edu/NielsenEtAl/. (4.3 MB ZIP). [file pbio.0020422.st001.zip › NielsenEtAl/html/91.html]

AN6630.1.NCU00635.1.MG02660.1.FG08560.1


```
 CLUSTAL W (1.82) Multiple Sequence Alignments - Introns Inserted


Sequence 1: MG02660.1	201 aa
Sequence 2: FG08560.1	209 aa
Sequence 3: NCU00635.1	200 aa
Sequence 4: AN6630.1	203 aa
Alignment Length: 215 aa
Number Identitical Residues: 115 aa
Alignment Score (without introns) 4948


MG02660.1 	MANPRVEELPDEEVKKTVVDDHDDDSSSDSDGEE--ETNLPAGSTAVIHSRNEKKARKAI
NCU00635.1	MADPRVEELPEEEVKKTQVEDLDNSSDDESDIEAG-DSSLPAGSQAVIHSRNEKKARKAI
FG08560.1 	MSNPRVEELPDEEPKKTTVQEHEDDSSDDSEVEEVGEGQLPAGST-VIHNRNEKKARKAL
AN6630.1  	MADPRIEELPDEEVPKTNVEDAADSS--ESEAGE--EPTIPGGAAVTIHSRNEKKARKAI
          	*::**:****:**  ** *::  :.*  :*:     :  :*.*:  .**.*********:

MG02660.1 	EKLHLIRVDGITRVTLRRPKN0ILFVINNPEVYKSPNSGTYI2VFGEAKIEDLNASAQAA
NCU00635.1	EKLHLQRVPGITRVTLRRPKN0ILFVINNPEVYKSPNSNTYI2VFGEAKIEDLNASAQAA
FG08560.1 	EKLHLTRIPGITRVTLRRPKN0ILFVINTPEVYKSPNSNTYI2VFGEAKIEDVNAAAQQA
AN6630.1  	GKLGLKHVPGITRVTLRRPKN0ILFVINQPDVYRSPSSNTWI2IFGEAKIEDLNSQAQAS
          	 ** * :: ************ ****** *:**:**.*.*:* :********:*: ** :

MG02660.1 	AAQQLASSAGHDHDHAGHSHGE----AKASEGDAKK--------EEEDDDEEVDADGIED
NCU00635.1	AAQQLASQS-AEHDHAGHTHEH----EEAGKAKEEE---------EEDEGEEVDAEGIED
FG08560.1 	AAAQLASQN--AEDHSGHNHGEP---SKAVEADEKKEDKEDDEDEEEEEEEEVDASGLED
AN6630.1  	AAQQLAAAE-AAGEHAGHDHDHDKGKGKAPETEAKKE-------EEEDDGEEVDETGLEP
          	** ***:      :*:** * . ... :* : . ::.       .**:: ****  *:* 

MG02660.1 	KDIELVMTQAGVSRTKAIKALKENDNDIVNSIMALSV
NCU00635.1	KDIELVMTQANVSRKKAIKALKENDNDIVNSIMALSI
FG08560.1 	KDIELVMTQANVSRNKAVKALKENDNDIVNSIMALSI
AN6630.1  	KDIDLVMAQANVSRKKAVKALRENDNDIVNSIMALSI
          	***:***:**.***.**:***:**************:
```
